# Phase Stability and Elasticity of TiAlN

**DOI:** 10.3390/ma4091599

**Published:** 2011-09-15

**Authors:** Igor A. Abrikosov, Axel Knutsson, Björn Alling, Ferenc Tasnádi, Hans Lind, Lars Hultman, Magnus Odén

**Affiliations:** Department of Physics, Chemistry, and Biology (IFM), Linköping University, Linköping 58183, Sweden; E-Mails: igor.abrikosov@ifm.liu.se (I.A.A.); knutsson@ifm.liu.se (A.K.); bjoal@ifm.liu.se (B.A.); tasnadi@ifm.liu.se (F.T.); halin@ifm.liu.se (H.L.); larhu@ifm.liu.se (L.H.)

**Keywords:** hard coatings, spinodal decomposition, *ab initio* calculations, thermodynamics, multilayer, TiN

## Abstract

We review results of recent combined theoretical and experimental studies of Ti_1−*x*_Al_*x*_N, an archetypical alloy system material for hard-coating applications. Theoretical simulations of lattice parameters, mixing enthalpies, and elastic properties are presented. Calculated phase diagrams at ambient pressure, as well as at pressure of 10 GPa, show a wide miscibility gap and broad region of compositions and temperatures where the spinodal decomposition takes place. The strong dependence of the elastic properties and sound wave anisotropy on the Al-content offers detailed understanding of the spinodal decomposition and age hardening in Ti_1−*x*_Al_*x*_N alloy films and multilayers. TiAlN/TiN multilayers can further improve the hardness and thermal stability compared to TiAlN since they offer means to influence the kinetics of the favorable spinodal decomposition and suppress the detrimental transformation to *w*-AlN. Here, we show that a 100 degree improvement in terms of *w*-AlN suppression can be achieved, which is of importance when the coating is used as a protective coating on metal cutting inserts.

## 1. Introduction

Transition metal carbides and nitrides belong to a class of materials with a unique combination of properties, high hardness, high melting point and excellent electrical conductivity, which make them attractive in many technological applications. Hard, wear-resistant, thin films based on this material family have been commercially available for some decades, and the business continues to expand rapidly. The self-organized nanostructured functional thin films are most often prepared by physical vapor deposition techniques followed by a thermal treatment [1]. Substantial efforts in this field are concentrated on the studies of growth processes, with considerable success. At the same time, a post-growth thermal treatment, as well as thermal and pressure conditions during the materials operation, substantially influence the film phase composition and microstructure. This is a quite general effect, which can either improve materials properties, or lead to detrimental effects limiting material performance. Therefore, there is a great need to fundamentally understand phase stability and its relation to physical properties, including the thermodynamics of alloy formation and decomposition, elasticity, and hardness.

In particular, spinodal decomposition is a common process of phase transformation for multicomponent systems in this family [2]. By the latter, one understands a phase transformation that takes place without nucleation and growth stage because an alloy is thermodynamically unstable rather than metastable. Specifically, any concentration fluctuation in such a material leads to its decomposition. In this article we will consider (Ti-Al)N alloy: a common system for modern hard coatings. Alloying of TiN with Al was suggested in order to make the coatings more resistant against oxidation [3,4,5,6,7,8,9] and the ternary nitride Ti_1−*x*_Al_*x*_N has become very popular in industrial applications. Synthesized by vapor deposition techniques under typical conditions, thin films of Ti_1−*x*_Al_*x*_N for *x* < 0.7 take the form of a cubic *B*1 (NaCl) structure substitutionally disordered alloys where one of the two sublattices is occupied by effectively randomly distributed Ti and Al atoms while the other sublattice is occupied by N atoms. It is important to underline that the system is not thermodynamically stable and, given that the amount of Al is large enough or that sufficient amount of thermal energy is supplied to the system, a decomposition into wurtzite(*w*) AlN and cubic(*c*) TiN or Ti enriched Ti_1−*x*_Al_*x*_N takes place. However, we suggested that a spinodal decomposition of this pseudobinary nitride system might play an important positive role in the age hardening of the coating. It has been shown to take place by the formation of coherent Ti-enriched *c*-Ti_1−*x*_Al_*x*_N regions and *c*-AlN nanograins [10,11,12].

**Figure 1:**
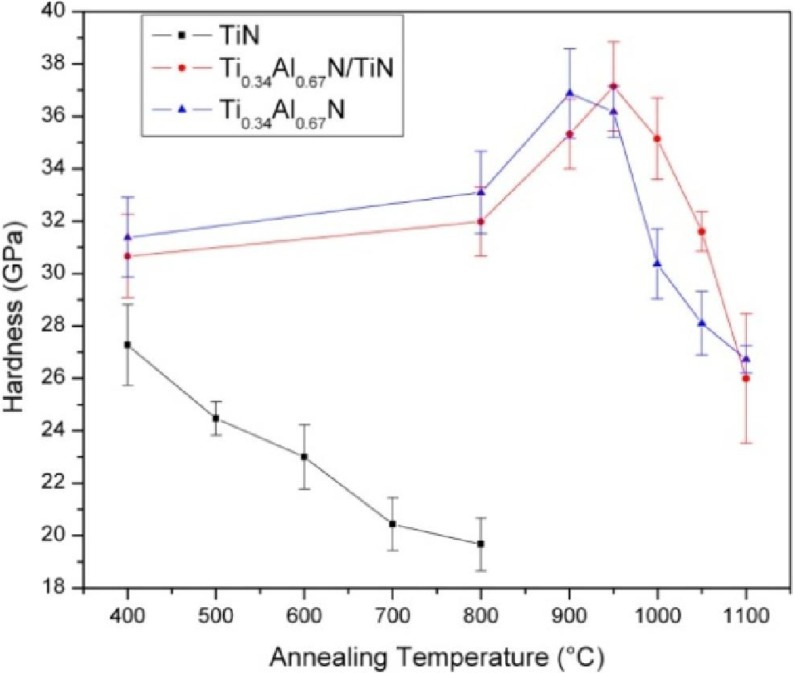
Hardness values obtained in [10] for the as-deposited and annealed monolithic TiN (black line with squares) and Ti_0.34_Al_0.66_N (blue, triangles), as well as multilayered Ti_0.34_Al_0.66_N/TiN coatings.

Figure 1 shows the *ex situ* hardness results of as deposited and isothermally heat treated samples of monolithic TiN and Ti_0.34_Al_0.66_N coatings, as well as multilayered Ti_0.34_Al_0.66_N/TiN coatings [10] to be discussed in details in Section 3. The TiN coating shows an overall decrease in hardness with increased annealing temperature. In contrast, Al-containing coatings show the qualitatively different appearance with an almost constant hardness up to 800 °С followed by an increase in hardness, associated with the age hardening due to the spinodal decomposition [11,12,13], and a decrease at high temperatures due to the cubic-to-wurtzite transformation of AlN.

In this article we review our recent theoretical and experimental work directed to the understanding of thermodynamic and elastic properties of Ti_1−*x*_Al_*x*_N alloys, the phase stability of this system, including the binodal and spinodal decomposition. We demonstrate that pressure from the applied force of a cutting tool against the work piece, together with the minimal contact area, has a beneficial effect, promoting the isostructural spinodal decomposition and suppressing the wurtzite phase formation. We also show the enhanced hardening phenomena and improved thermal stability of the multilayer coatings, and discuss the effect in terms of particle confinement and coherency stresses from the neighboring TiN-layers.

## 2. Properties of Ti_1−*x*_Al_*x*_N Alloys from *ab Initio* Simulations

The dominating approach in studying materials is experiment. However, at present the electronic structure theory allows one to obtain reliable results for the thermodynamic, mechanical, electrical and magnetic properties of metals, semiconductors or insulators without any adjustable parameters fitted to the experiment [14]. Of course, until recently this was mostly done for relatively simple systems (for example, a perfectly ordered compound). At the same time, we can see today that the first-principles simulations are extended towards more realistic materials, which are of direct importance for practical applications.

In particular, an efficient first-principles method that can be used to calculate electronic and thermodynamic properties of transition metal nitrides with B1 structure and substitutional disorder at the metal sublattice has been developed by Alling *et al.* [15]. The technique is based on the density functional theory and the exact muffin-tin orbital method (EMTO), which allows for treatment of the substitutional disorder within the coherent potential approximation (CPA) [16]. The independent sublattice model allows for the treatment of disorder-induced local lattice relaxation effects, often neglected in the CPA, but essential for Ti_1−*x*_Al_*x*_N [15]. It is developed essentially to account for the effect of the relaxation of the nitrogen and metal atoms relative to each other in the *B*1-nitride case. Together with the effective tetrahedron method, it has been used for the description of local lattice relaxations on the transition metal sublattice, giving highly accurate relaxation energies, in excellent agreement with fully-relaxed supercell calculations [15]. The efficiency of the EMTO-CPA technique allows for total energy calculations on a very fine mesh of concentrations which enables a reliable calculation of the second concentration derivative of the alloy total energy. This is an essential quantity, which, when below zero, determines the tendency of an alloy towards the spinodal decomposition. In reference [15], electronic structure, lattice parameter, and mixing enthalpies of the quasibinary Ti_1−*x*_Al_*x*_N alloys were calculated.

Another approach used for the theoretical description of the substitutional disorder on transition metal sublattice in Ti_1−*x*_Al_*x*_N is the supercell technique [15,17]. The so-called Special Quasirandom Structure (SQS) method [15,18,19] gives carefully designed supercells that match the distribution of alloy components in the random alloy on the average for a number of nearest neighbors shells with non-vanishing effective cluster interactions. The SQS approach is easily combined with state-of-the-art full potential techniques for the electronic structure calculations, like Vienna *Ab Initio* Simulation Package (VASP) [20,21,22] used for the majority of simulations presented here. As was demonstrated in [15], the two techniques agree very well with each other, so we refer to the original publications concerning which of them was used for a particular calculation in this review.

### 2.1. Basic Thermodynamic Properties of Ti_1−x_Al_x_N: Lattice Parameters and Mixing Enthalpies

**Figure 2:**
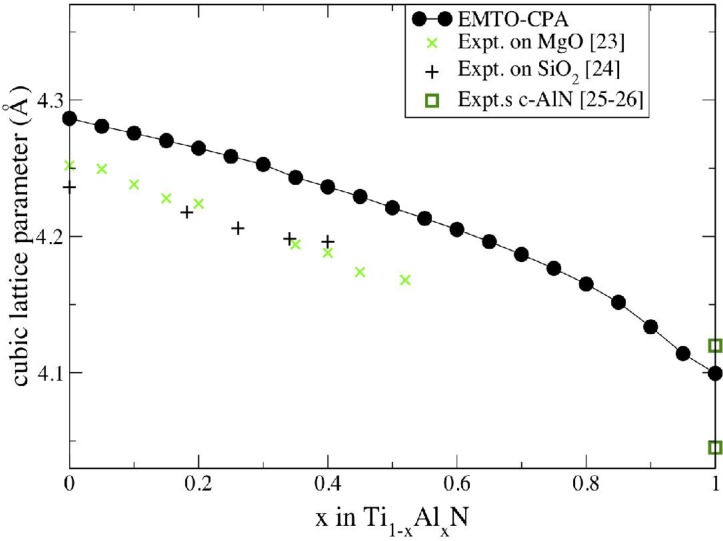
The lattice parameter of Ti_1−*x*_Al_*x*_N as a function of the fraction of AlN, *x*, obtained from EMTO-CPA calculations (filled circles) [15]. The experimental results from references [23,24,25,26] are shown for comparison.

Figure 2 demonstrates the calculated lattice parameter as a function of fraction of AlN. One sees that the calculated values are in good agreement with the experimental data [23,24,25,26]. The value for pure TiN (4.29 Å) overestimates the experiment by about 1%, which is common for *ab initio* calculations. As a matter of fact, using full-potential VASP calculations, the agreement can be further improved [15]. The calculated value for pure *c*-AlN (4.10 Å) is between the two experimental values from references [25] and [26], respectively. Importantly, the theory convincingly reproduces the experimental trends: there is a relatively small size mismatch between the end members in this system and a close to linear dependence of the lattice constant on AlN fraction *x* at small values of *x*, with increasing deviation from Vegard’s law at higher *x*. This observation is important for the discussion of the effect of pressure on the phase stability of Ti_1−*x*_Al_*x*_N alloys below.

**Figure 3:**
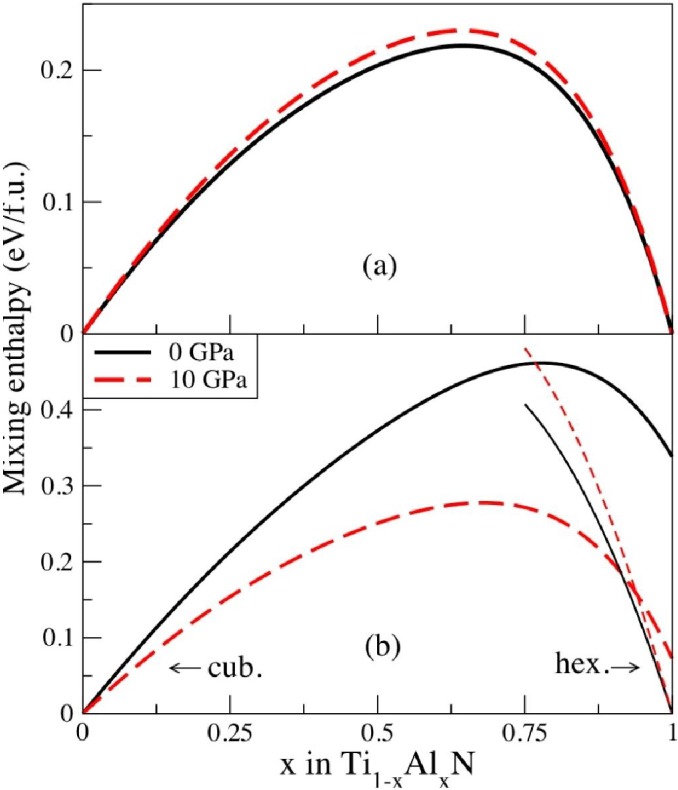
(**a**) Isostructural cubic mixing enthalpy of Ti_1−x_Al_x_N at pressures P = 0 GPa and P = 10 GPa; (**b**) Mixing enthalpy of cubic rock salt (thick lines) and hexagonal wurtzite (thin lines) Ti_1−x_Al_x_N as a function of AlN fraction x at pressures P = 0 GPa and P = 10 GPa, relative to cubic TiN and hexagonal AlN.

One of the key quantities that determines the alloy phase stability is the mixing enthalpy $H_{Ti_{1-x}Al_{x}N}^{mix}$, defined as:

(1) $$H_{Ti_{1-x}Al_{x}N}^{mix}(P,x)=H_{Ti_{1-x}Al_{x}N}(P,x)-(1-x)H_{TiN}(P)-xH_{AlN}(P)$$

where $H_{Ti_{1-x}Al_{x}N}$, $H_{TiN}$, and $H_{AlN}$ are the enthalpies of the alloy and end members compounds, TiN and AlN, respectively, all calculated at the same pressure *P*. Figure 3 shows this quantity, calculated in reference [27] at ambient pressure and at pressure 10 GPa. In panel (**a**) the so-called isostructural mixing enthalpy is presented, calculated with respect to cubic phases of TiN and AlN, while in (**b**) we show the mixing enthalpy, calculated with respect to *c*-TiN and *w*-AlN. The latter defines the overall stability of the alloy, while the former governs the stability of the solid solution with respect to isostructural decomposition, essential for the study of the spinodal decomposition. Large positive values of the isostructural mixing enthalpy indicate that the alloy is highly unstable with respect to decomposition into *c*-TiN and *c*-AlN. Even higher positive values in Figure 3(**b**) show strong preference for the decomposition into *c*-TiN and *w*-AlN. However, it can be suppressed due to elastic and kinetic effects, since the barriers for the isostructural decomposition should be much smaller.

**Figure 4:**
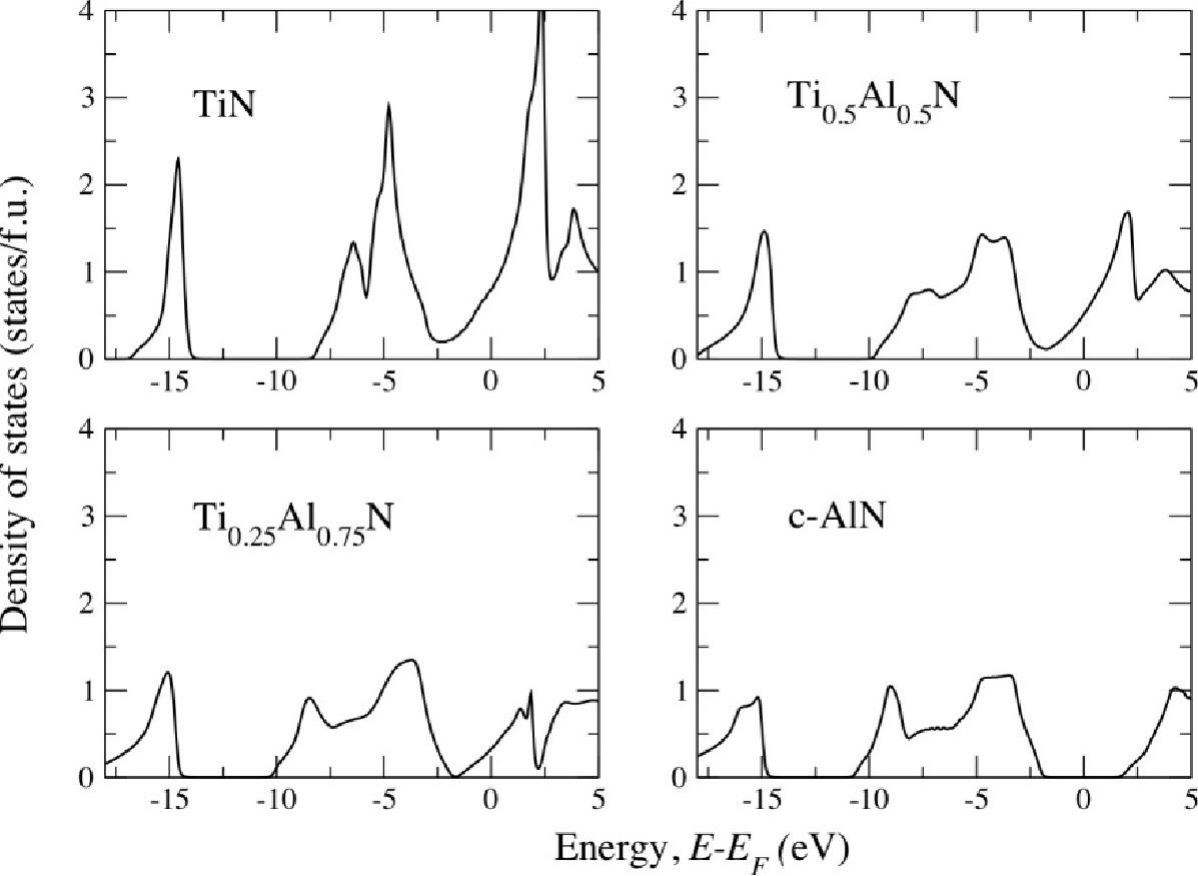
Total electronic density of states (DOS) for c-Ti_1−x_Al_x_N calculated in [15] as a function of energy (relative to Fermi energy E_F_) calculated for different fractions x of AlN (0.00, 0.50, 0.75, and 1.00) shows the presence of the metal-to-insulator transition when one goes from metalic TiN to semiconducting AlN.

From Figure 3(**a**) one can also see that the isostructural mixing enthalpy of Ti_1−*x*_Al_*x*_N exhibits rather complicated behavior. It is highly asymmetric with respect to the equiatomic composition. This effect cannot be captured by a regular solution model or its simple generalizations, and needs to be carefully accounted for in, e.g., thermodynamic simulations of TiAlN system using phenomenological thermochemical approaches. Such behavior of the system, and the changes of its thermodynamic properties with concentration, is related to a gradual electronic structure transition from metallic TiN to semiconductor AlN, which actually is the metal-to-insulator transition as illustrated in Figure 4. In particular, a substitution of Ti with Al leads to a cutting of the next-to-nearest neighbor (metal sublattice nearest-neighbor) Ti-Ti bonds of t_2*g*_ character, and eventually leads to isolated and localized Ti t_2*g*_ states in a semiconducting AlN-rich matrix [15]. This observation is also highly important for the determination of the effective cluster interactions in this system, responsible for the configurational thermodynamics of alloys [19,28]. We will return to this problem in Section 2.3.

From Figure 3(**a**) we also conclude that the pressure makes the isostructural mixing enthalpies more positive. Thus, one should expect an increase of the tendency towards the isostructural decomposition under compression [27]. However, the change is relatively small, the maximum value is increased from 0.22 eV/f.u. at 0 GPa to 0.23 eV/f.u. at 10 GPa. In contrast, a much stronger effect of the compression is seen in the non-isostructural case, Figure 3(**b**). The results indicate a gradual stabilization of the cubic phase in the AlN rich region due to a decrease in the mixing enthalpy [27]. The crossing point of the enthalpies of the cubic and hexagonal phases shifts from *x* = 0.71 at 0 GPa to *x* = 0.94 at 10 GPa. This observation is in agreement with calculations by Holec *et al.* [29], and also in line with the fact that cubic *B1* AlN is stabilized by pressure with respect to its wurtzite phase [30]. This finding indicates that detrimental cubic-to-wurtzite transformation of AlN-rich domains is less likely to occur during for example cutting operations where the TiAlN coating is exposed to a combination of heat and pressure than in an annealing experiment carried out at ambient conditions.

### 2.2. Decomposition Thermodynamics of Ti_1−x_Al_x_N at Ambient and Elevated Pressure

Upon a consideration of the alloy thermodynamics, one deals with a system in thermal and mechanical contact with a constant-temperature constant-pressure heat bath, whose equilibrium is described by the thermodynamic potential

*G = E + PV − TS = H – TS = F + PV* (2)

where *G* is the Gibbs free energy, *E* is the internal energy of the system, *H* is the enthalpy, *S* denotes the entropy, *F* represents the Helmholtz free energy *F = E − TS*, and thermodynamic variables *P*, *V*, *T* represent pressure, volume, and temperature, respectively. For the phases in equilibrium at constant *P* and *T* in a quasi-binary alloy, like Ti_1−x_Al_x_N, the *G* curves as a function of fraction of one of the end member compound (AlN in our case) x must share a common tangent. Moreover, if

(3) $$\frac{\partial^{2}G}{\partial x^{2}}<0$$

a system becomes unstable to any fluctuation of composition, and a spinodal decomposition can take place if diffusion allows it to.

The first-principles simulations of the phase stability of alloys are based on the atomistic description of the problem. The thermodynamic potential is most often the Helmholtz free energy *F*, however, there is no problem to recalculate *G* from Equation (1). For the canonical ensemble the Helmholtz free energy is calculated as:

(4) $$F(T,V,N)=-k_{B}T\text{ln}Z(T,V,N)=-k_{B}T\text{ln}\left[\sum_{\left\{R\right\}}\text{exp}\left(-\frac{E_{\left\{R\right\}}}{k_{B}T}\right)\right]$$

where *Z(T,V,N)* is the partition function and *k_B_* is the Boltzmann constant. In Equation (4) the sum runs over all possible states *{R}* of the system (for example, if we need to find the lowest energy configuration for Ti_1−x_Al_x_N alloy at fixed AlN fraction *x* and B1 underlying crystal lattice, *{R}* represents all possible occupations of the fcc transition metal sublattice by *(1−x)N* Ti and *xN* Al atoms).

A determination of energies *E_{R}_* in Equation (4), generally speaking, requires a solution of a complex many-body quantum mechanical problem for each particular atomic configuration *{R}* of the solution phase. To avoid this impossible task, it is most appropriate to consider atomic configurations *{R}* at the sites of an underlying crystal lattice. In this case the alloy energetics can be described by the Ising Hamiltonian:

(5) $$E_{\left\{R\right\}}=V^{\left(0\right)}+\sum \left[V^{\left(1\right)}\langle \sigma \rangle +\sum_{s}V^{\left(2,s\right)}\langle \sigma_{i}\sigma_{j}\rangle +...\right]$$

where *i,j* are lattice sites, the spin variables *σ_i_* takes on values +1 or −1 depending on the type of atom-occupying site *i*. The average products of the spin-variables, <*σ_i_σ_j_*>, are the multisite correlation functions which form the complete basis for the total energy expansion [19], *V^(0)^* is the reference energy, which, in fact, is the total energy of a random equiatomic alloy, and *V^(d,s)^* are the effective cluster interactions, which correspond to clusters of the order *d* and type *s*. For instance, $V^{\left(2,1\right)}=V_{AA}^{nn}+V_{BB}^{nn}-2V_{AB}^{nn}$ is the effective pair (*d* = 2) interaction at the first coordination shell (*s* = 1), which describes interactions between all the different types of pairs (AA, BB, and AB) of nearest neighbor (nn) atoms in the A_1−x_B_x_ alloy. The on-site interaction *V^(1)^*, which is the effective chemical potential, can be neglected in the canonical ensemble calculations (that is, if the number of particles in a simulation box is conserved).

**Figure 5:**
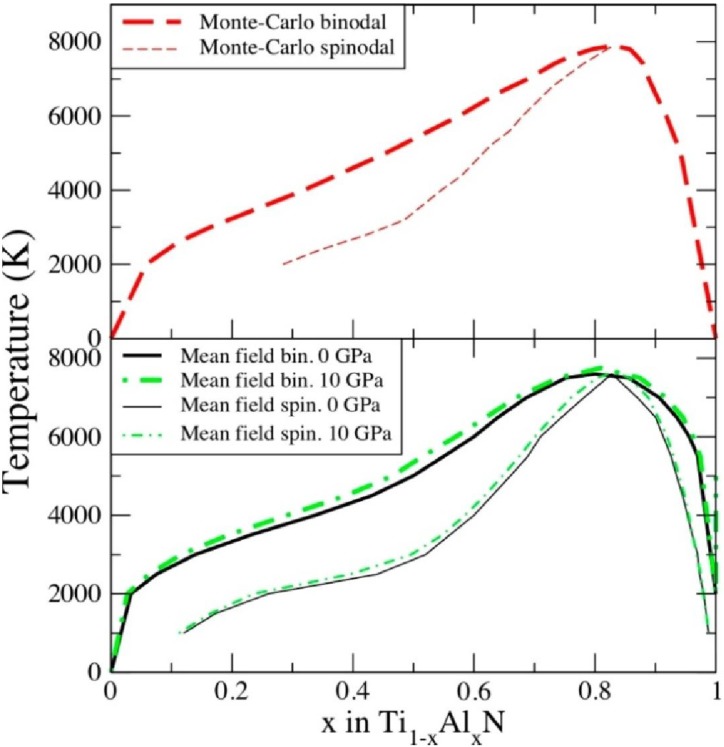
The isostructural phase diagram of cubic Ti_1−x_Al_x_N as calculated with the Monte Carlo approach [28] (**a**) and with the mean-field approximation [15,28] (**b**). In the latter case we also show the phase diagram calculated at elevated pressure P = 10 GPa [27]. The binodal lines are shown with thick lines while the spinodal lines are shown by thin lines.

The coarse-graining procedure in going from quantum mechanical description to classical description consists of the determination of parameters in Equation (5), the effective cluster interactions *V* on the basis of first-principles DFT calculations. A number of different procedures exist for extracting many-body interactions for alloys out of electronic structure calculations, and they are reviewed in reference [19]. However, neither of them could be safely used for Ti_1−x_Al_x_N because of the metal-to-insulator transition, Figure 4, and large contribution due to local lattice relaxations [15]. Thus, in reference [28], a new, so-called unified cluster expansion has been developed. The purely configurational part of the alloy Hamiltonian has been expanded in terms of concentration and volume-dependent effective cluster interactions. Separate expansions are done of the chemical fixed lattice and local lattice relaxation terms of the ordering energies. The novelty of the approach in [28] is the fruitful combination of the screened generalized perturbation method with a concentration-dependent Connolly-Williams cluster expansion method, giving rise to the unified cluster expansion. Utilizing the so determined interactions in Monte Carlo simulations combined with the thermodynamic integration, the free energy *G* of Ti_1−x_Al_x_N alloy has been calculated and the isostructural phase diagram has been constructed. The results are presented in Figure 5a. They show striking similarities with the mean-field results (Figure 5b) obtained earlier in reference [15], the metastable c–TiAlN is subject to spinodal decomposition over a larger part of the concentration range, e.g., from x ≥ 0.33 at 2000 K [15]. This justifies the use of this much more efficient method in studies of decomposition thermodynamics of Ti_1‑x_Al_x_N.

Employing the mean-field description, the effect of pressure on the decomposition thermodynamics of TiAlN has been studied in reference [27]. The cubic isostructural phase diagram, which is essential for a discussion of the spinodal decomposition, is shown in Figure 5(**b**). One can see that, in agreement with mixing enthalpies calculations in Figure 3(**a**), the tendency towards decomposition, binodal, as well as spinodal, increases. This can be understood from the following thermodynamics arguments [27]. The pressure derivative of the Gibb’s free energy at fixed temperature

(6) $${\left(\frac{\partial G}{\partial P}\right)|}_{T}=V$$

For the free energy of mixing

(7) $$G_{Ti_{1-x}Al_{x}N}^{mix}(P,x)=G_{Ti_{1-x}Al_{x}N}(P,x)-(1-x)G_{TiN}(P)-xG_{AlN}(P)$$

one therefore obtains

(8) $${\left(\frac{\partial G_{Ti_{1-x}Al_{x}N}^{mix}}{\partial P}\right)|}_{T}=\text{Δ}V$$

where

(9) $$\text{Δ}V=V_{Ti_{1-x}Al_{x}N}-(1-x)V_{TiN}-xV_{AlN}$$

is the deviation of the alloy volume from Zen’s law [31]. From Figure 2, one sees that it is positive in Ti_1-x_Al_x_N, thus explaining the results in Figure 5(**b**).

As expected, the effect of pressure up to 10 GPa on the topology of the phase diagram is not large. However, it is seen that, due to a distinct shoulder at compositions just below x = 0.50, even relatively small pressures can substantially increase the spinodal region at a given temperature. Note that the applied force of a cutting tool against the work piece, together with the minimal contact area, gives rise to stress or pressure levels of several GPa at the cutting edge [32]. Also, common cathode arc-deposition processing for TiAlN yields coatings with a few GPa intrinsic compressive stress due to residual lattice defects [1]. Thus, the observation above should be included in a consideration of the coating performance. In particular, the spinodal decomposition is believed to lead to the age hardening effect (Figure 1). Together with a suppression of cubic-to-wurtzite transformation in AlN-rich alloys, as discussed in Section 2.1, the overall effect of pressure should be beneficial for the applications of Ti_1−x_Al_x_N-based coatings.

### 2.3. Elastic Properties of Ti_1−x_Al_x_N Solid Solutions

The spinodal decomposition is influenced by elastic anisotropy [33], and the hardness enhancement observed upon the age hardening relies on a shear modulus difference between the formed domains [34] as well as their coherency strain (see below). Thus, it is of primary interest to understand the elastic properties of Ti_1−x_Al_x_N alloys, and consequently, first principles calculations of elastic constants for this system have been carried out [35,36]. In this section we discuss the theoretical modeling of the influence of composition x in the Ti_1−x_Al_x_N system on the elastic constants and elastic anisotropy.

For the accurate calculations of elastic constants in reference [35], full-potential calculations were carried out with VASP code [20,21,22] and the disorder was modeled using 96 atoms (4 × 4 × 3) supercell constructed as the SQS [18,19]. The SQS was generated by optimizing the Warren-Cowley pair short-range order (SRO) parameters [37] on the Ti-sublattice up to the 7th neighboring shell. The elastic stiffness constants were derived from the second order Taylor-expansion coefficients of the total energy:

(10) $${C_{ij}=\frac{1}{V_{0}}\frac{\partial^{2}E(\varepsilon_{1},...,\varepsilon_{6})}{\partial \varepsilon_{i}\partial \varepsilon_{j}}|}_{0}$$

where Voigt’s notation is used to describe the strain tensor ε. In the calculations ±1% and ±2% deformations to the lattice parameters of the supercells we employed, and finite difference technique was used to derive the values of the stiffness constants.

**Figure 6:**
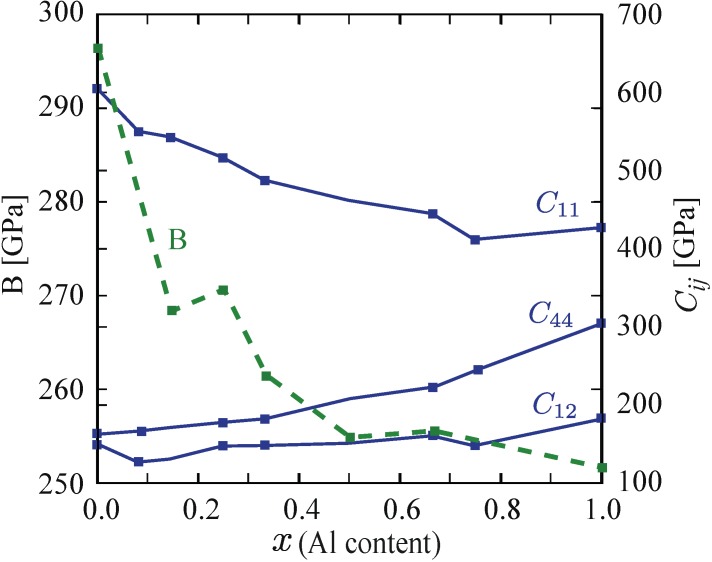
Calculated bulk modulus *B* and elastic stiffness constants, *C_11_*, *C_12_*, and *C_44_*, of c-Ti_1−x_Al_x_N as a function of fraction *x* of AlN [35].

Figure 6 shows the obtained *ab-initio* bulk moduli, *B*, as well as the cubic elastic constants, *C_11_*, *C_12_*, and *C_44_* of Ti_1−x_Al_x_N alloys over the entire compositional range. Generally, they behave smoothly along the line pointed out by the parent binary compounds, c-AlN and c-TiN. *C_12_* and *C_44_* increase with the amount of Al concentration, while *C_11_* and *B*, in comparison, show a pronounced decrease. The increase of *C_44_* and the decrease of *B* have also been theoretically observed in supercell calculations for Ti_1−x_Al_x_N in reference [36].

The concentration behavior of the elastic constants has important implications on the general elastic behavior of the system, as the longitudinal sound velocity *v_hkl_* has been shown [35] to decrease in the (100) direction and increase in (111). The strong increase of *v_111_* is unambiguously connected to the increasing *C_44_* in Figure 6, while the dropping *v_100_* is a clear consequence of the softening of *C_11_*.

**Figure 7:**
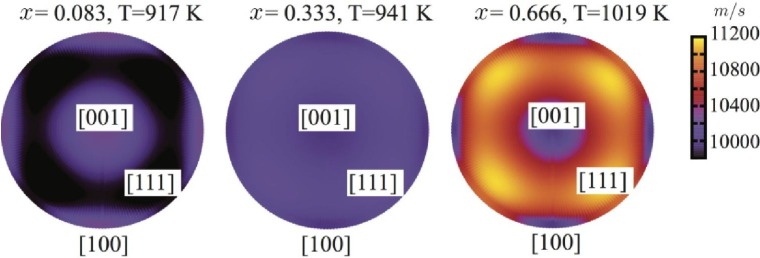
Calculated longitudinal sound velocity anisotropy map and Debye temperatures for c-Ti_1−x_Al_x_N at different functions *x* of AlN. The surface plots show the calculated spherical distribution (θ,φ) of the longitudinal sound velocity from top view assigned by high symmetry directions.

To quantify the elastic anisotropy in this system, we invoke the analysis of acoustic waves anisotropy via the Christoffel equation [38]. Our results are shown in Figure 7. They indicate elastic isotropy in cubic Ti_1−x_Al_x_N around *x* = 0.33, but significant anisotropy of qualitatively different topology at lower and higher compositions, respectively. The directional anisotropy of the longitudinal sound velocities indicates significant difference in the nature of the nearest neighbor bonds with increasing Al-content.

## 3. Improved Thermal Stability from Multilayered Architecturing of Ti_1−x_Al_x_N Alloys

Having established theoretically the wide range of composition and temperatures, where Ti_1−x_Al_x_N alloys undergo the spinodal decomposition, we deal in this section with the age hardening and the kinetics of thermal decomposition of Ti_0.34_Al_0.66_N and how these phenomena are affected by a multilayer architecture, *i.e*., the size of transforming material and influence of epitaxy with the neighboring layers. A multilayer coating with 25 and 50 nm thick Ti_0.34_Al_0.66_N and TiN layers respectively were grown using a commercial Sulzer/Metaplas MZR-323 reactive cathodic arc evaporation system. For more details regarding the multilayer deposition, see reference [10]. The idea is to utilize the fact that, as discussed in Section 2, TiN and c-AlN are immiscible (Figure 3 and Figure 5), but still have a lattice mismatch small enough (Figure 2) to allow coherency across the multilayer interfaces. Further, the TiN is in a thermal equilibrium and will not interfere with the thermal responses from the Ti_0.34_Al_0.66_N layers [39]. Multilayer coating structures have proven to exhibit hardening [40] due to the significant composition dependence of elastic constants of Ti_1−x_Al_x_N alloys (Figure 6), leading to shear modulus differences between the layers [34] and coherency strain at the internal interfaces [41]. Differential scanning calorimetry has been used successfully to study the thermal responses during annealing and to clarify how the thermal stability is affected by a multilayer structure with coherent interfaces. Analytical scanning transmission electron microscopy (STEM) with energy dispersive spectroscopy (EDX) and X-ray diffraction (XRD) were used for microstructure characterization and nanoindententation for mechanical property determination of as-deposited and *ex-situ* 120 min annealed coatings [10].

### 3.1. Thermal Stability of Monolithic Ti_0.34_Al_0.66_N

X-ray diffraction data from monolithic Ti_0.34_Al_0.66_N on WC-Co substrate in its as-deposited state show peaks originating only from c-Ti_0.34_Al_0.66_N [10]. In agreement with the theoretical phase diagram in Figure 5, the diffractogram from the coating heat treated at 900 °C shows a decrease of the diffraction peak intensities from fcc-Ti_0.34_Al_0.66_N and the appearance of c-TiN and c-AlN which are due to the spinodal decomposition. Heat treatment at 1000 °C results in a decrease of the c-AlN peak intensity and the appearance of w-AlN, which is a result of the transformation from c-AlN to w-AlN. After heat treatments at 1100 °C no change of the w-AlN intensity is seen, suggesting that the decomposition is at an end already at 1000 °C.

**Figure 8:**
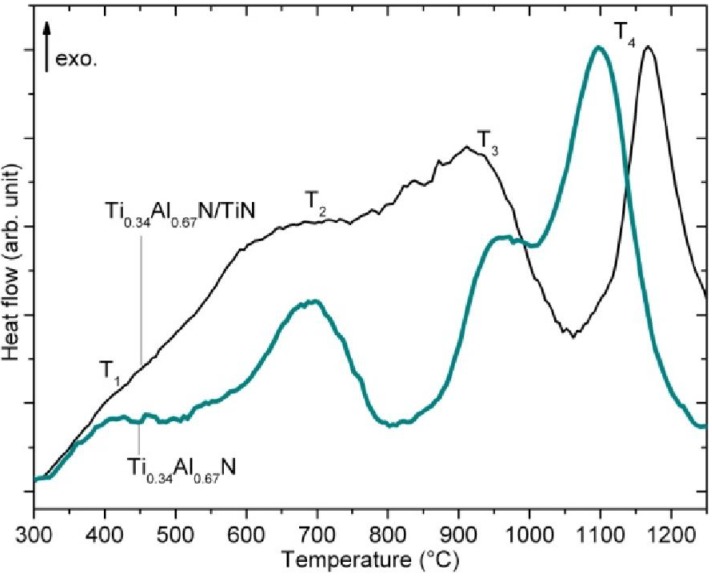
Differential scanning calorimetry measurements of the monolithic and multilayered coating [10].

Figure 8 shows the heat flow responses of monolithic Ti_0.34_Al_0.66_N [10,39]. The thermogram shows four peaks positioned at T_1_~400 °C, T_2_~700 °C, T_3_~960 °C, and T_4_~1097 °C. The peaks labeled T_1_ and T_2_ corresponds to recovery processes of lattice point defect complexes with different activation energies induced during deposition. This effect is expected at temperatures higher than the deposition temperature of 400 °C [10,42]. The peak labeled T_3_ is related to the isostructural spinodal decomposition in the c-Ti_0.34_Al_0.66_N layers and it occurs in the 800–1000 °C regime. This is supported by the occurrence of c-TiN and c-AlN phases in XRD measurements at the same temperature. Note, that according to theoretical calculations of the phase diagram, Figure 5, the alloy is still deep inside the spinodal region. Thus, there is on-set of the spinodal decomposition as the temperature is high enough for atomic diffusion. This is not the case at lower temperatures for which the decomposition is suppressed by kinetics, even though the alloy is inside the spinodal region.

The next peak, T_4_ located in the 1097–1187 °C regime corresponds to the c-AlN to w-AlN transformation. This is the final step of transformation and the original c-Ti_0.34_Al_0.66_N will past this step consist dominantly of c-TiN and w-AlN, which is again in accordance with XRD measurements.

### 3.2. The Multilayer Structure Influence on the Thermal Stability of Ti_0.34_Al_0.66_N

**Figure 9:**
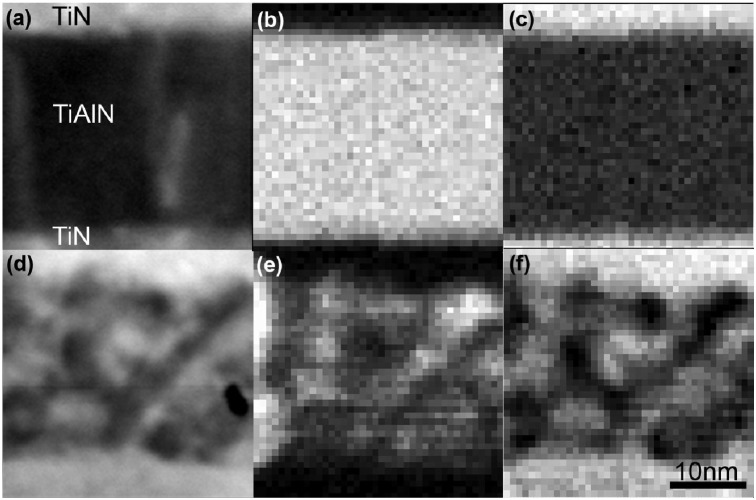
STEM images of as-deposited sample (**a**) and 900°C annealed sample (**d**) and corresponding Al and Ti STEM-EDX elemental maps in as deposited state (**b**)–(**c**) and annealed state (**e**)–(**f**) [10].

X-ray diffractometry on the as-deposited multilayer coating shows that it is a polycrystalline Ti_0.34_Al_0.66_N/TiN multilayer with distinctly different peaks from the two different cubic phases and has a 002 preferred orientation. [10,43]. STEM-EDX elemental maps of a single Ti_0.34_Al_0.66_N layer in its as-deposited state, Figure 9 (a–c) show an overall homogenous distribution of Al and Ti [43]. Based on the homogeneous distribution of elements, the lack of Al in the interfacing TiN layers, and the fact that only XRD peaks from TiN and Ti_0.34_Al_0.66_N exist for the as-deposited multilayer, it is concluded that the individual layers in the multilayer consist of alternating single phase TiN and Ti_0.34_Al_0.66_N. The coating heat-treated at 900 °C exhibits, as in the monolithic case, peaks corresponding to metastable c-AlN. The positions of the Ti_0.34_Al_0.66_N peaks also reveal a relative increase in TiN content due to the apparent phase separation.

STEM imaging and elemental mapping of the coating annealed at this temperature, Figure 9 (e–f), reveal that the Ti_0.34_Al_0.66_N has decomposed to domains of high Al content surrounded by areas of low Al and high Ti content. The size of the domains of the decomposed structure has been confirmed with small angle X-ray scattering [44] and atom probe tomography [45]. High resolution TEM micrographs of the Ti_0.34_Al_0.66_N layer in as-deposited and heat treated at 900 °C states show lattice coherency within the layer and across layer interface, consistent with a spinodal decomposition mechanism, predicted by theoretical simulations at these conditions in Section 2. Heat treatments at 1000 °C result in a decrease of the 002 c-AlN peak and increase of all TiN and the w-AlN peaks. Heat treatments at 1100 °C result in, contrary to the monolithic TiAlN, a further increase of the 1010 w-AlN peak intensity.

Figure 8 shows the thermal responses of the multilayer coating compared to the monolithic coating. The graph has the same basic appearance as the graph corresponding to the monolithic coating, with four overlapping but distinct peaks. The location of the T_1_ and T_2_ peaks appear to be the same for both the multilayer and the monolithic coating and they appear in a narrow interval of only ~8 °C. A distinct difference between the monolithic coating and the multilayer is however seen for peak T_4_ where the multilayer peak response is shifted to a higher temperature with 90 °C. Hence, the DSC data from the multilayers indicate that the formation of w-AlN is suppressed in the multilayer structured compared to the monolithic coating. This is also supported by the X-ray diffraction where the intensity from the w-AlN, as mention, was reported to increase between 1000 and 1100 °C in the multilayered coating while it is constant for the monolithic coating. In addition, the onset of spinodal decomposition, peak T_3_, occurs at slightly lower temperatures for the multilayers.

### 3.3. Mechanical Stability of TiAlN

Let us now return to Figure 1. It shows the *ex-situ* hardness measurements of as-deposited and isothermally heat treated samples of monolithic TiN and Ti_0.34_Al_0.66_N. The Ti_0.34_Al_0.66_N shows a slight increase of hardness up to 800 °C where a more pronounced increase in hardness (age hardening) is observed [11,12]. This is followed by a drastic decrease in hardness at high temperatures. As discussed in Section 2, strain fields as a result of the coherency, molar volume difference [30] and differences in elastic modules between c-TiN and c-AlN, that effectively hinder dislocation motions are reported to be the mechanism of the age hardening [11,12,35]. The decrease in hardness at 950 °C is related to the decomposition of metastable c-AlN into stable w-AlN. The transformation is accompanied by a ~20 % unit cell volume increase and a loss of the coherency and thus increase the possibility of dislocations movements [30,46,47]. The hardness of monolithic TiN, which is shown as a reference in Figure 1, decreases to its intrinsic hardness of approximately 20 GPa [48,49] due to defect annihilation [50].

### 3.4. Multilayer Structure Influence on Mechanical Stability of Ti_0.34_Al_0.66_N

Figure 1 shows also the hardness of the multilayered Ti_0.34_Al_0.66_N compared to the monolithic Ti_0.34_Al_0.66_N. In the as-deposited state the coating show a hardness similar to the monolithic Ti_0.34_Al_0.66_N, probably due to multilayer hardening effects [34]. Such hardening has been observed for many other transition metal nitride systems with elastic shear modulus differences [51,52]. A hardness of 35 ± 1.2 GPa for the multilayer is measured after annealing at 1000 °C which should be compared to 30 ± 1.1 GPa for the monolithic coating after the same heat treatment. This is a significant improvement difference of the hardness especially when considering that the multilayer consists of 60 vol.% TiN. Hence, a combination of multilayer hardening and Orowan-like strengthening inside the decomposing layers is suggested as the underlying mechanisms of the higher hardness, but also an improved thermal stability [51,53,54,55]. Accordingly, the DSC measurements explain the retained hardness of the multilayered Ti_0.34_Al_0.66_N coating. Since the transformation is evidently suppressed to higher temperatures in the multilayer, see peak T_4_ in Figure 8, the decrease in hardness occurs at higher annealing temperatures.

### 3.5. Effects on Alloying Elements and Nitrogen Vacancies on the Phase Stability of TiAlN

The phase stability as well as the mechanical properties of TiAlN coatings are effected, and might be enhanced, by alloying with additional elements and/or deliberate modifications of the nitrogen stoichiometry. In reference [56] it was shown by theoretical calculations that a small amount of nitrogen vacancies in Ti_1−x_Al_x_N tended to enhance the coherent isostructural decomposition of Ti and Al atoms on the metal sublattice. The reason was that cubic AlN showed a strong resistance against nitrogen off-stoichiometry due to its semiconducting, large bandgap electronic structure, while TiN could easily accommodate them. Thus, a possible clustering of nitrogen vancancies in Ti-rich regions gives an additional thermodynamic driving force for metal-sublattice decomposition.

Finally, the performance of TiAlN-based coatings can be further improved via alloying with additional elements. Influence of Nb and Ta on the phase stability of TiAlN has been considered in references [57,58]. Alloying TiAlN with Zr was found to promote the formation of cubic domains, but retard the formation of stable wurtzite AlN during thermal annealing, as well as to increase the hardness of the system [59]. Structure and mechanical properties of TiAlN-WN_x_ thin films were investigated in reference [60]. In reference [61] the concept of muticomponent alloying has been introduced as a design route for the next generation of nitride alloys. It is based on self-organization on the nanoscale via a formation of metastable intermediate products during the spinodal decomposition. It was predicted theoretically and demonstrated experimentally that quasi-ternary (TiCrAl)N alloys decompose spinodally into (TiCr)N and (CrAl)N-rich regions, which are only few nanometers large. The spinodal decomposition resulted in age hardening, while the presence of Cr within the AlN phase delayed the formation of a detrimental wurtzite phase leading to a substantial improvement of thermal stability compared to both the corresponding parent nitrides, as well as quasi-binary alloys.

## 4. Conclusions

We have analyzed results of recent studies of Ti_1−x_Al_x_N alloys, and demonstrated the power of a combined theoretical and experimental approach for obtaining a fundamental understanding of the properties of this hard material. Great potential is presented for materials designed by coating layer architecture and composition as well as thermal and load processing during cutting application.

Calculated lattice parameters of Ti_1−x_Al_x_N show positive deviations from Vegard’s and Zen’s laws, which explains the influence of pressure on the phase stability in this system. The strong asymmetry of the mixing enthalpy is shown to be due to electronic structure effects, the presence of the metal-to-insulator transition. The advanced technique for taking the latter effect into consideration upon the simulation of the phase diagram of B1 cubic Ti_1−x_Al_x_N was developed. Monte-Carlo and mean-field calculations of the phase diagram are in good agreement with each other. The system is predicted to be within the unstable, spinodal regime over a large composition range at all relevant temperatures. Pressure is predicted to increase the tendency towards the isostructural binodal and spinodal decomposition, while suppressing the formation of detrimental wurtzite phase of AlN. Theoretical modeling of the influence of Al content in the Ti_1−x_Al_x_N alloy on the elastic constants and elastic anisotropy, which also agrees well with experiment, suggests that the latter is strongly affected by the composition and therefore is an important parameter for tailoring the properties of hard coatings.

We present evidence of an improved thermal stability of the Ti_0.34_Al_0.66_N in a multilayer structure compared to a monolithic Ti_0.34_Al_0.66_N coating. Our results show that the spinodal decomposition is shifted to lower temperatures while the precipitation of w-AlN is shifted to higher temperatures. Further control of the phase stability and secondary phase transformations in TiAlN-based coatings may be achieved by additional alloying and/or nitrogen stoichiometry.
